# Molecular characterization of directly reprogrammed neurons from human fibroblasts using single cell RNA sequencing

**DOI:** 10.1038/s41598-025-34688-8

**Published:** 2026-01-09

**Authors:** Do-Jin Seo, Jin-Ah Kim, Jung Ho Lee, Yoon-Ho Hong

**Affiliations:** 1 Neuroscience Research Institute, Seoul National University College of Medicine, Seoul, Republic of Korea; 2 Genomic Medicine Institute, Medical Research Center, Seoul National University, Seoul, Republic of Korea; 3 Department of Chemistry, Seoul National University, Seoul, Republic of Korea; 4 Department of Neurology, Medical Research Council, Seoul Metropolitan Government-Seoul National University Boramae Medical Center, Seoul, Republic of Korea

**Keywords:** Cellular reprogramming, Polypyrimidine Tract-Binding protein 1, Single-Cell analysis, Cell differentiation, Molecular neuroscience, Gene regulatory networks

## Abstract

Direct neuronal reprogramming offers a promising strategy to generate functional neurons from somatic cells without passing through a pluripotent state. However, the cellular heterogeneity and molecular transitions underlying this process remain incompletely understood in human cells. Here, we transduced human dermal fibroblasts with lentiviral short hairpin RNA targeting polypyrimidine tract-binding protein 1 (PTBP1) and performed single-cell RNA sequencing (scRNA-seq) at day 14 post-transduction. PTBP1 knockdown was associated with neurogenic transcription factor upregulation and the emergence of neuronal morphology, with conversion efficiency reaching approximately 20% by week 2. scRNA-seq revealed a progression from fibroblasts toward transcriptionally distinct immature and mature neuronal populations, and pseudotime analysis provided a descriptive ordering of cells along divergent transcriptional gradients that reflect transitions toward either neuronal or myofibroblast-like states. Using an analytic framework designed to ensure statistical robustness in single-sample data, we identified transcription factors that reliably distinguish mature from immature neuronal lineages. These findings provide mechanistic insight into barriers to neuronal maturation and highlight potential molecular targets that may improve the efficiency of direct neuronal reprogramming in human cells.

**Supplementary Information:**

The online version contains supplementary material available at 10.1038/s41598-025-34688-8.

## Introduction

Advances in disease modelling play a critical role in understanding complex pathophysiological mechanisms and guiding the development of effective therapies^1^. Patient-derived cell models can be particularly valuable, as they may provide insights directly relevant to human biology. However, obtaining such models for neurodegenerative diseases remains challenging due to the limited availability of human brain tissue samples. Traditional approaches such as cell culture, animal models and stem cell-based technologies such as embryonic stem cells (ESCs) and induced pluripotent stem cells (iPSCs), present notable limitations, including species-specific differences, ethical concerns, and technical challenges^2–4^.

Direct neuronal reprogramming has emerged as a promising alternative for generating functional neurons from patient-derived somatic cells without passing through an intermediate pluripotent state^5,6^. Unlike iPSC-based differentiation, which requires reprogramming into a pluripotent state before neuronal induction, direct neuronal reprogramming bypasses this step by inducing neuronal identity through forced expression of transcription factors, microRNAs (miRNAs) or RNA-binding proteins^7,8^. *PTBP1* has garnered significant interest for its regulatory influence on various RNA processing events, particularly in alternative splicing and microRNA biogenesis relevant to neurogenesis^9,10^. Recent findings demonstrate that modulating *PTBP1* levels is crucial for facilitating developmental pathways that lead to functional neuron formation from somatic cells^11,12^.

Despite advances in direct neuronal reprogramming, the molecular and cellular heterogeneity of reprogrammed cells, and the regulatory networks governing this process remain incompletely understood^13–15^. Single cell RNA sequencing (scRNA-seq) enables a comprehensive analysis of gene expression profiles at the individual cell level, facilitating the identification of distinct neuronal progenitor states and maturation trajectories. This high-resolution approach is particularly relevant, as neuronal conversion often elicits diverse cellular responses that traditional bulk analysis might obscure^16^. Here, to induce direct neuronal reprogramming, we transduced human dermal fibroblasts with a lentiviral short hairpin RNA targeting *PTBP1*. The transcriptomic profiles of the reprogrammed cells were subsequently analyzed using scRNA-seq, to investigate the transcriptional dynamics that underpin changes in cellular identity and the mechanisms that govern neuronal differentiation and maturation.

## Results

### Direct neuronal conversion of human skin fibroblasts via ***PTBP1*** downregulation

**Fig. 1:**
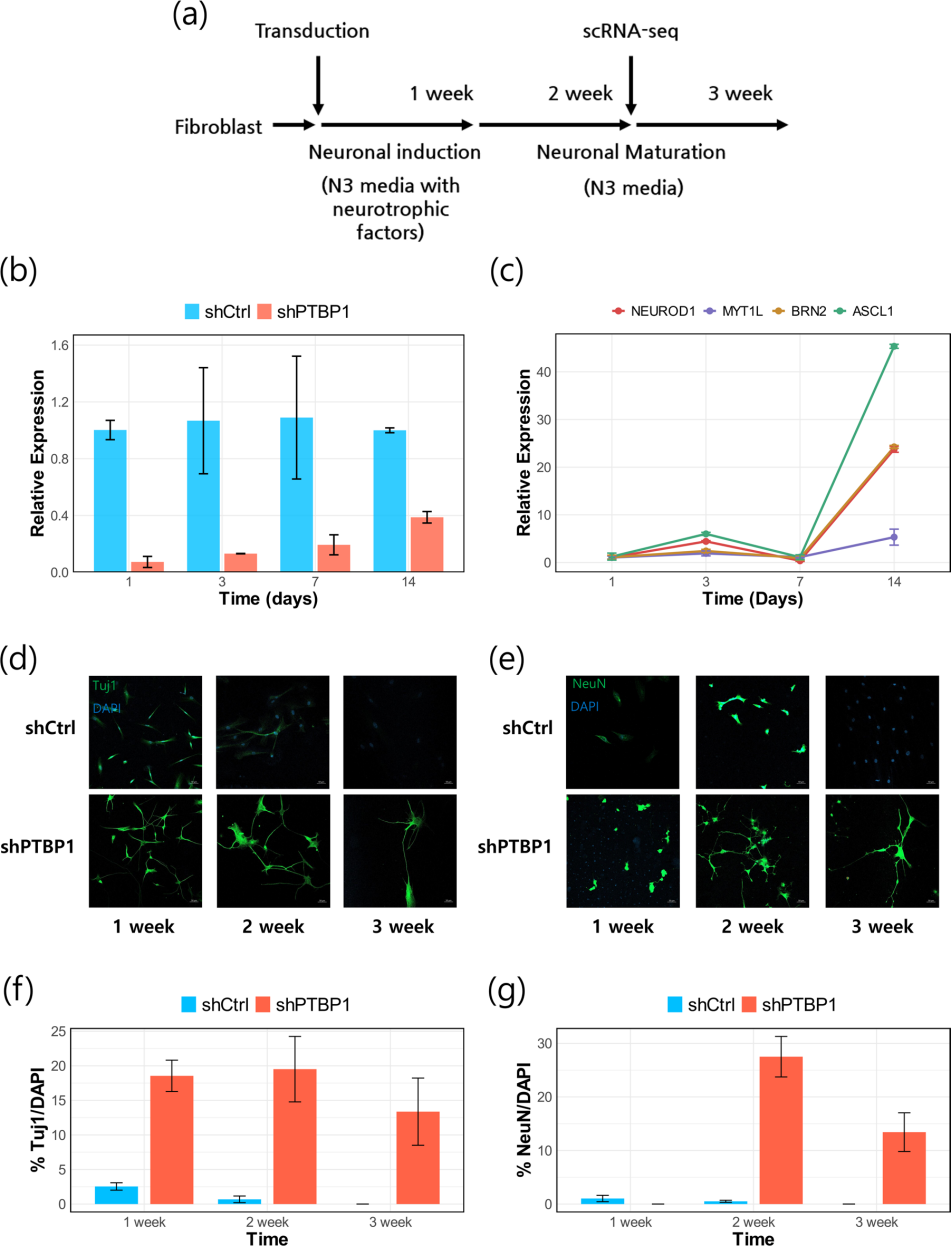
PTBP1 downregulation promotes neuronal differentiation in human skin fibroblasts. (**a**) A schematic representation of the reprogramming process for generating induced neurons (iNs) from fibroblasts using lentiviral shPTBP1 transduction. (**b**) Relative *PTBP1* mRNA expression in shCtrl vs. shPTBP1 groups over time, measured by qPCR. Two-way repeated measures ANOVA revealed a significant main effect of Treatment (*p* < 0.01), while the effects of Time and Treatment × Time interaction were not significant (*p* > 0.05). (**c**) Time-course analysis of transcription factor expression (*ASCL1*,* NEUROD1*,* BRN2*, and *MYT1L*) following shPTBP1 treatment. One-way repeated measures ANOVA with Bonferroni correction showed significant temporal changes in all factors except Myt1l (*p* < 0.01). (**d**, **e**) Immunofluorescence staining for Tuj1 (**d**) and NeuN (**e**) at 1, 2, and 3 weeks post-transduction confirmed neuronal differentiation. Representative images were selected to highlight neuron-like morphology, including the presence of long neurites and well-defined somatic contours, rather than to reflect average fluorescence intensity across all imaged fields. Scale bars, 50 μm. (**f**, **g**) Quantification of DAPI-positive cells co-expressing Tuj1 (**f**) and NeuN (**g**) in shCtrl and shPTBP1 groups over time. For Tuj1, two-way repeated measures ANOVA revealed a significant main effect of Treatment (*p* < 0.01), with no significant effects of Time or the Treatment × Time interaction (*p* > 0.05). For NeuN, significant main effects were observed for both Treatment and Time (*p* < 0.01), along with a significant Treatment × Time interaction (*p* < 0.01), indicating that temporal changes differed depending on treatment condition. Error bars represent SEM.

To initiate neuronal conversion, human dermal fibroblasts were transduced with lentiviral shPTBP1, as illustrated in Fig. 1a. Quantitative RT-PCR confirmed efficient knockdown of *PTBP1*, which was sustained through day 7 and partially restored by day 14 (Fig. 1b). In parallel, neurogenesis-associated transcription factors—including *ASCL1*,* BRN2*,* MYT1L*, and *NEUROD1*—were upregulated, with Ascl1 exhibiting the most robust increase (Fig. 1c).

Immunofluorescence analysis revealed early morphological and molecular changes consistent with neuronal differentiation, including neurite extension and βIII-tubulin (Tuj1) expression as early as day 7, and NeuN expression by day 14 (Fig. 1d, e). Notably, NeuN exhibited predominantly cytoplasmic localization in these early-stage reprogrammed neurons, consistent with reported patterns in differentiating and cultured neuronal systems^17–19^. This distribution pattern was independently validated in differentiated SH-SY5Y neuroblastoma cells using the identical antibody, confirming that cytoplasmic NeuN localization reflects the immature neuronal state of our reprogrammed cells rather than technical artifact (Supplementary Fig. 5). In contrast, fibroblasts transduced with control shRNA (shCtrl) did not exhibit neuronal morphology and remained negative for both Tuj1 and NeuN.

Quantification of neuronal marker expression demonstrated a time-dependent increase in the proportion of induced neurons (iNs), peaking at day 14 with approximately 20% of DAPI-positive cells co-expressing NeuN (Fig. 1f, g). These results indicate that *PTBP1* downregulation promotes neuronal conversion in human dermal fibroblasts, although the overall efficiency remains limited. Based on these findings, we performed transcriptomic profiling at day 14 to further investigate the molecular mechanisms underlying direct neuronal reprogramming.

#### Single-Cell RNA-seq analysis reveals cellular heterogeneity and differentiation trajectories

**Fig. 2:**
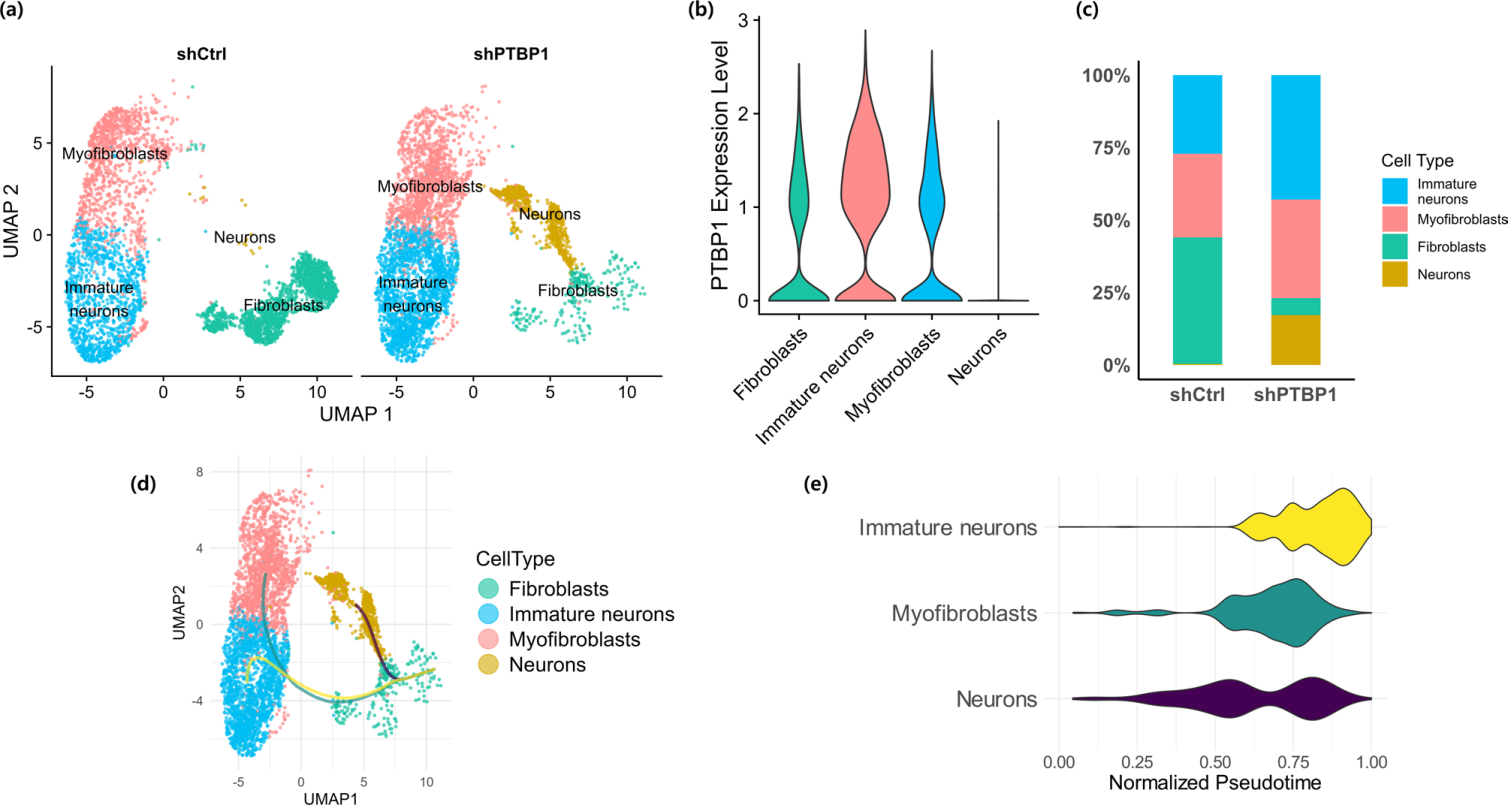
Single-cell RNA sequencing reveals cellular heterogeneity and differentiation trajectories following ***PTBP1*** knockdown. (**a**) UMAP visualization of scRNA-seq data showing annotated cell types in shCtrl and shPTBP1 groups. Neuronal populations (neurons and immature neurons) are increased in the shPTBP1 group, while fibroblast-like populations are decreased. (**b**) Violin plots of *PTBP1* expression in the shPTBP1 group. Expression is markedly reduced in neurons. (**c**) Cell type composition in shCtrl and shPTBP1 groups, showing a marked reduction in fibroblasts and a corresponding increase in immature neurons and mature neuronal populations following PTBP1 knockdown. (**d**) Lineage trajectory analysis using Slingshot reconstructs differentiation pathways to neurons, immature neurons and myofibroblasts. (**e**) Violin plots show the distribution of normalized pseudotime for immature neurons, myofibroblasts, and neurons inferred from trajectory analysis within the shPTBP1 condition. The bimodal distribution observed for the neuronal lineage indicates the predominance of discrete transcriptional states rather than a continuous differentiation trajectory.

To examine the transcriptional dynamics underlying *PTBP1* downregulation-mediated neuronal reprogramming, scRNA-seq was performed at day 14 post-transduction using dermal fibroblasts treated with either shPTBP1 or shCtrl. Cell type annotation was conducted using the platform, which integrates marker databases and cell-type classification algorithms^20^. In the shPTBP1 group, four major cell populations were identified: fibroblasts, myofibroblasts, immature neurons, and neurons (Fig. 2a). In contrast, the shCtrl group primarily comprised fibroblasts and myofibroblasts, with minimal neuronal conversion.

To confirm the efficiency of *PTBP1* knockdown, we assessed *PTBP1* expression across annotated cell types, which showed markedly reduced *PTBP1* expression in neurons and immature neurons, confirming successful *PTBP1* knockdown in these cell populations (Fig. 2b). Quantification of cell type proportions further indicated a decrease in fibroblast-like cells and an increase in neuronal populations in the shPTBP1 group, although the overall neuronal differentiation efficiency remained relatively low (~ 20%) (Fig. 2c).

To characterize cluster-specific gene expression profiles, we performed differential gene expression analysis using the *FindAllMarkers* function in Seurat. This function identifies genes that are significantly upregulated in each cluster compared to all other clusters. We restricted the analysis to positive markers (only.pos = TRUE), with a minimum log2 fold change of 0.25 and expression in at least 20% of cells (min.pct = 0.2), using the Wilcoxon rank sum test. From this analysis, the top 10 marker genes for each cluster were selected based on adjusted p-values and fold changes (Supplementary Table 2). Canonical markers such as glutamate ionotropic receptor NMDA type subunit 2 A (*GRIN2A*) for glutamatergic neurons and gamma-aminobutyric acid type B receptor subunit 2 (*GABBR2*) for GABAergic neurons were identified, supporting the cluster annotations. Clusters annotated as immature neurons exhibited elevated expression of cell cycle–related genes (e.g., *NUF2*,* UBE2C*), suggesting ongoing proliferation. Cell type annotation was further validated by comparing canonical marker gene expression across clusters (Supplementary Fig. 2). These findings support the biological identity of each cluster and validate the automated annotations based on known markers.

To clarify the neuronal subtype composition, we characterized the expression of canonical markers using both transcriptomic and immunofluorescence approaches. *GRIN2A* and *GABBR2* were differentially expressed across distinct neuronal subclusters, indicating the presence of glutamatergic and GABAergic neurons, respectively (Supplementary Fig. 3a). These subtype identities were further confirmed by immunostaining for vGlut1 and GABA-T (Supplementary Fig. 3b). For subsequent analyses, the glutamatergic and GABAergic neuronal subclusters were combined and collectively referred to as “neurons” to distinguish them from the immature neuronal population.

To substantiate our findings beyond single-gene markers, we performed systematic gene set enrichment analysis using the SynGO database^21,22^. To address potential ontology selection bias and confounding by ubiquitous structural genes (e.g., myofibroblasts), we implemented the rigorous CountSplit method^23^. This approach ensures statistical validity by computationally thinning sequencing data into two independent partitions, thereby separating gene selection from hypothesis testing. In the exploratory partition (Split A), we identified 145 SynGO-annotated genes specifically upregulated in neurons relative to myofibroblasts (log2FC > 0.2, adjusted p-value < 0.05). When this refined gene set was applied to the independent validation partition (Split B), the ‘Neurons’ cluster exhibited significantly higher AUCell enrichment scores compared to ‘Immature neurons’ and ‘Myofibroblasts’ (*p* < 0.001, pairwise Wilcoxon test, Supplementary Fig. 4). Cross-validation (Selection on Split B, Inference on Split A) yielded consistent results.

To investigate potential lineage relationships, pseudotime trajectory analysis was performed using Slingshot, which arranges cells along inferred differentiation paths based on transcriptional similarity. Given the single-timepoint design, pseudotime ordering was interpreted as a descriptive arrangement of transcriptionally distinct populations along maturation-related gene expression gradients, rather than as evidence of temporal progression^24^. Fibroblasts were assigned as the root population, and three terminal clusters were desginated based on prior annotations set to represent neurons, immature neurons, and myofibroblasts. This analysis identified transcriptionally distinct paths connecting fibroblasts to neuronal or myofibroblast states (Fig. 2d), representing possible reprogramming routes that warrant validation through time-resolved studies. Notably, UMAP-based clustering revealed that immature neurons and myofibroblasts occupy adjacent positions in low-dimensional space, despite being transcriptionally distinct lineages. This spatial proximity suggests that some cells in this region may represent a partially reprogrammed hybrid state. These cells co-express early neuronal markers (tubulin alpha 1c (*TUBA1C*)) and myofibroblast-associated genes (actin alpha 2, smooth muscle (*ACTA2*)), supporting the interpretation that they have not fully committed to either fate.

Pseudotime distribution along the neuronal lineage revealed a bimodal pattern, suggesting that while a subset of fibroblasts efficiently transitioned into neurons early during reprogramming, others stalled in an immature neuronal state (Fig. 2e).

## Trajectory-based differential expression analysis

Next, we examined transcriptional differences underlying incomplete neuronal maturation by comparing the dynamics of cells differentiating into mature versus immature neurons. The trajectory terminating in the neuronal population was designated as the “mature neuronal lineage,” representing cells that successfully completed neuronal conversion. Neuronal subtypes were merged into a single group for trajectory analysis, as our goal was to characterize global transcriptional programs associated with successful versus incomplete reprogramming rather than subtype-specific developmental processes.

To ensure statistically valid inference under the single-replicate design, we implemented the Countsplit method^23^ with bidirectional cross-validation. Trajectory inference and differential expression testing were performed in both directions:

Path 1: trajectory on Split A, DE on Split B; Path 2: trajectory on Split B, DE on Split A.

**Fig. 3:**
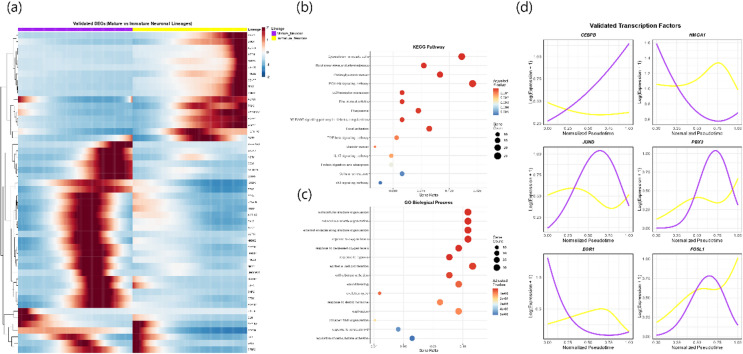
Bidirectionally validated trajectory-based differential expression analysis. (**a**) Heatmap of the top 50 genes from the 342 bidirectionally validated differentially expressed genes distinguishing mature versus immature neuronal lineages. Genes were selected using patternTest on an independent split in the Countsplit framework. (**b**) KEGG pathway enrichment of the 342 validated genes, highlighting ECM–receptor interaction, PI3K–Akt signaling, cytoskeletal regulation, and proteoglycan-related pathways. Dot size denotes gene count and color indicates adjusted p-values. (**c**) GO Biological Process enrichment showing extracellular matrix organization, oxygen- and hypoxia-response pathways, wound healing, and cell–substrate adhesion. (**d**) Pseudotime expression profiles of the six validated transcription factors demonstrating lineage-specific dynamics along the mature (purple) and immature (yellow) neuronal trajectories.

Path 1 identified 360 genes (FDR < 0.05), and Path 2 identified 360 genes. The final 342 genes consistently significant in both directions were retained as the validated gene set, ensuring robustness to split assignment. Differential expression analysis was performed with the patternTest function in tradeSeq^25^. Given the single late-stage sampling and discrete intermediate states, pseudotime ordering was interpreted as a descriptive arrangement of transcriptionally distinct populations along maturation-related gradients rather than evidence of temporal progression. Heatmap clustering of the 342 validated genes revealed distinct expression modules (Fig. 3a shows the top 50 ranked by effect size). Functional enrichment analyses showed that these validated genes are strongly associated with extracellular matrix remodeling, cytoskeletal reorganization, and stress-response pathways. KEGG pathway enrichment^26^ highlighted terms such as ECM–receptor interaction, PI3K–Akt signaling, cytoskeletal regulation, and proteoglycan-related pathways (Fig. 3b). GO Biological Process enrichment further identified extracellular matrix organization, hypoxia and oxygen-response pathways, wound healing, and cell–substrate adhesion (Fig. 3c). Together, these profiles reflect the substantial structural and adaptive changes required for fibroblasts to transition toward neuronal identity.

To identify potential regulatory drivers of the divergence between mature and immature neuronal lineages, validated genes were screened against the DoRothEA transcription factor database^27,28^. transcription factors (CEBPB, EGR1, PBX3, HMGA1, JUND, and FOSL1) passed stringent Countsplit validation (FDR < 0.05). Their pseudotime expression profiles showed distinct lineage-specific dynamics along the mature (purple) and immature (yellow) trajectories (Fig. 3d), suggesting coordinated regulatory roles in neuronal fate specification and maturation.

## Discussion

In this study, we evaluated the ability of PTBP1 downregulation to induce direct neuronal conversion in human dermal fibroblasts. Although PTBP1 knockdown promoted neuronal-like morphological and molecular features, only a subset of cells progressed to a mature neuronal state, underscoring the limited efficiency of the reprogramming process. To investigate the molecular basis for this heterogeneity, we applied single-cell transcriptomic profiling together with trajectory-based analysis using Slingshot and tradeSeq. This integrative approach enabled a descriptive ordering of transcriptional transitions and revealed distinct gene expression programs separating mature from immature neuronal populations. Pseudotime analysis showed a bimodal distribution of neuronal-lineage cells, with mature neurons enriched at earlier pseudotime positions and immature neurons occupying later positions. This pattern suggests that cells with higher reprogramming competence complete neuronal maturation, whereas others stall in an immature state. These findings imply that while PTBP1 downregulation initiates neuronal commitment, additional regulatory mechanisms are likely required for full maturation.

To explore these mechanisms, we identified transcription factors associated with divergence between mature and immature neuronal states using DoRothEA annotations combined with bidirectional CountSplit validation. This analysis yielded six transcription factors with statistically validated differential expression patterns (FDR < 0.05): CEBPB, EGR1, PBX3, HMGA1, JUND, and FOSL1. These validated regulators displayed lineage-specific pseudotime expression dynamics and likely contribute to complementary aspects of neuronal development. Functionally, these factors can be grouped into three categories. First, CEBPB, EGR1, and PBX3 have established roles in neuronal specification and maturation: CEBPB in hippocampal neurogenesis and neural stem cell differentiation, EGR1 as an immediate-early gene mediating activity-dependent plasticity, and PBX3 in neuronal subtype specification^29–31^. Second, HMGA1 acts as a chromatin remodeling factor influencing neurogenic potential and the timing of neuronal differentiation^32^. Third, JUND and FOSL1, members of the AP-1 family, regulate transcriptional programs and chromatin accessibility involved in cell state transitions^33,34^. Their coordinated, lineage-specific expression patterns suggest that transcriptional, epigenetic, and chromatin architectural mechanisms collectively shape reprogramming trajectories.

These transcription factors are likely indirect downstream effects of PTBP1 downregulation rather than immediate targets, consistent with the temporal delay between PTBP1 knockdown and transcription factor upregulation observed in our time-course analysis (Fig. 1b–c). Their lineage-specific dynamics indicate that a coordinated, stage-dependent regulatory program is necessary to achieve full neuronal maturation. The validated factors identified here thus represent testable candidates for improving reprogramming efficiency through combinatorial perturbation.

The relatively low conversion efficiency in human cells contrasts with results from mouse models, where PTBP1 downregulation alone is sufficient to induce neuronal reprogramming^11^. This interspecies discrepancy suggests that human reprogramming involves more complex, multi-step regulatory processes shaped by distinct transcriptional and epigenetic landscapes. Recent studies report that additional factors, such as PTBP2, are required to promote neuronal reprogramming in human systems^10,35^. The delayed upregulation of neurogenic transcription factors such as ASCL1 and NEUROD1 may reflect multi-step progression within the reprogramming cascade, as previously described8. PTBP1 knockdown induces a transient increase in PTBP2 (nPTB), followed by progressive derepression of neuronal gene networks; once activated, these transcription factors form self-reinforcing regulatory loops that maintain neuronal gene expression even as PTBP1 levels begin to recover. Epigenetic remodeling and establishment of a permissive chromatin landscape may further contribute to the observed temporal dynamics of neurogenic gene activation. Together, these findings underscore the importance of species-specific optimization when designing reprogramming strategies.

From a methodological standpoint, this study illustrates both the utility and limitations of trajectory-based single-cell analysis in reprogramming research. The transcriptional heterogeneity in the shPTBP1 group at day 14 enabled inference of pseudotemporal organization using Slingshot; however, these trajectories should be interpreted as descriptive transcriptional gradients, rather than validated temporal progressions. Slingshot infers lineage structure from cluster topology rather than chronological sampling, allowing hypothesis generation from cross-sectional data^36,37^. When combined with tradeSeq, this framework facilitated high-resolution mapping of transcriptional transitions and highlighted lineage-specific regulatory programs.

Several limitations warrant consideration. Canonical neuronal protein markers such as Tuj1 and NeuN were not robustly detected in our scRNA-seq dataset despite clear immunofluorescence signals. This discrepancy is consistent with known limitations of single-cell transcriptomics, including transcript dropout—particularly for low-abundance or transient transcripts—and the mismatch between mRNA abundance and protein stability in differentiating neurons^38,39^. Additionally, because this study relied on a single biological replicate per condition, findings should be viewed as exploratory and hypothesis-generating. Multiple biological replicates and pseudobulk-based modeling will be necessary to validate these results. Furthermore, the modest neuronal conversion efficiency (~ 20%) and the discrete separation of cell populations impose constraints on trajectory inference. Under these conditions, pseudotime orderings represent qualitative models of potential transcriptional transitions rather than definitive developmental sequences. Although we employed CountSplit to obtain statistically validated differential expression results despite single-replicate design, the identified transcription factors remain correlational. Functional perturbation and time-resolved profiling will be essential to establish causal roles in neuronal maturation.

In conclusion, we systematically characterized transcriptional heterogeneity during PTBP1-mediated direct neuronal reprogramming of human fibroblasts. Our analysis reveals a bifurcation in transcriptional transitions, with only a portion of cells progressing toward mature neuronal identity. By identifying six statistically validated transcription factors associated with divergence between immature and mature neuronal trajectories, we provide testable molecular targets for improving reprogramming outcomes. Although based on a single time point, the transcriptional diversity of the reprogrammed population enabled reconstruction of potential neuronal trajectories, offering insights into reprogramming heterogeneity. Future time-resolved studies incorporating multiple biological replicates and functional perturbations will be necessary to validate these inferred transitions and refine strategies to achieve mature, functional neuronal identity in human cells.

## Methods

### Ethics approval

This study was approved by the local institutional review board (IRB No. 30–2023-58). The study participant gave written informed consent. All methods were performed in accordance with the relevant guidelines and regulations.

### Fibroblast isolation

Primary human skin fibroblasts were isolated from a skin biopsy. A round-tipped hollow scalpel was used to obtain a 3 mm punch biopsy sample extending to the subcutaneous adipose tissue layer. Isolated fibroblasts were then cultured in Dulbecco’s Modified Eagle Medium (DMEM) supplemented with 20% fetal bovine serum (FBS), non-essential amino acids, sodium pyruvate, and penicillin/streptomycin.

## Lentiviral particles containing ShRNA targeting human PTBP1

Lentiviral short hairpin RNAs (shRNA) targeting human PTBP1 (TRCN0000231420, sequence: CCGGGCGTGAAGATCCTGTTCAATACTCGAGTATTGAACAGGATCTTCACGCTTTTTG) were obtained from Thermo Scientific and subcloned in the pLKO.1 vector for stable expression. Individual shRNA-containing plasmids were co-transfected with lentiviral packaging plasmids (Sigma) into HEK293T cells, generating replication-incompetent lentiviral particles containing the specific shRNA. Supernatant containing these viral particles was collected 48 h post-transfection for downstream applications.

## Cellular transduction and neuronal induction

Individual fibroblast cultures were transduced with lentiviral particles targeting *PTBP1* for 16 h. Infection was performed in fibroblast media supplemented with 8 µg/ml polybrene to enhance viral integration. Following transduction, cells were exposed to 1 µg/mL puromycin for 30 h to eliminate non-transduced cells and achieve stable shRNA expression. Following puromycin selection, selected cells were cultured in N3 media for 3 days to initiate neuronal differentiation. N3 media consisted of DMEM/F12 supplemented with insulin (25 µg/ml), transferrin (50 µg/ml), sodium selenite (30 nM), progesterone (20 nM), putrescine (100 nM), and fibroblast growth factor 2 (FGF2; 10 ng/ml).

### Immunofluorescence staining

Following differentiation, cells were washed with PBS and fixed with 4% paraformaldehyde for 20 min at room temperature. After three washes with PBS, cells were blocked with a protein-blocking solution (GBIlabs) for 30 min at room temperature. Primary antibodies specific for neuronal markers were then applied: Tubulin β 3 (TUBB3) (BioLegend, 801213, 1:1000), MAP2 (Sigma-Aldrich, M9942, 1:1000), VGLUT1 (Invitrogen, 48–2400, 1:1000), Choline Acetyltransferase (Sigma-Aldrich, AB144P, 1:100), Tyrosine Hydroxylase (Novus Biologicals, NB300-109, 1:1000), ABAT/GABA-T (Abcam, ab216465, 1:100), NeuN (Sigma-Aldrich, MAB377, 1:100). These antibodies were diluted in an antibody diluent (Life technologies) containing 0.2% Triton X-100 (Sigma) to promote membrane permeabilization and incubated for 20 h. Secondary antibodies conjugated to Alexa Fluor 488 and 594 (Thermo Fisher scientific, 1:5000) were diluted in PBS and applied for 1 h.

### SH-SY5Y differentiation and NeuN localization validation

To validate NeuN antibody specificity and confirm cytoplasmic localization patterns observed in reprogrammed neurons, we used SH-SY5Y human neuroblastoma cells (ATCC CRL-2266) as a positive control for cultured neuronal systems. SH-SY5Y cells were maintained in DMEM/F12 (Gibco) supplemented with 10% fetal bovine serum, 100 U/ml penicillin, and 100 µg/ml streptomycin at 37 °C in 5% CO₂. For neuronal differentiation, cells were seeded at 2 × 10⁴ cells/cm² and differentiated using a two-step protocol: cells were first treated with 10 µM all-trans retinoic acid (Sigma-Aldrich, R2625) in serum-reduced medium (1% FBS) for 7 days to initiate neuronal commitment, followed by medium replacement with serum-free DMEM/F12 containing 50 ng/ml brain-derived neurotrophic factor (BDNF; PeproTech, 450-02) for an additional 7 days to promote neuronal maturation. Medium was changed every 2–3 days throughout the differentiation period. Differentiated SH-SY5Y cells were processed for immunofluorescence using the identical staining protocol and anti-NeuN antibody (Sigma-Aldrich MAB377, clone A60, 1:100) as described above for reprogrammed fibroblasts. This served as an independent validation of NeuN localization patterns in cultured neuronal cells.

### Quantification of neuronal induction efficiency

Neuronal induction efficiency was determined as follow^40^. At 14 days post-transduction, at least 15 randomly selected microscopic fields per condition were analyzed to assess the proportion of induced neurons. Cells were identified as induced neurons based on neuronal morphology and positive immunostaining for Tuj1 or NeuN. For each image, the number of Tuj1-positive, NeuN-positive, and DAPI-positive cells was counted. The percentage of neuronal induction was calculated by dividing the number of Tuj1- or NeuN-positive cells by the total number of DAPI-positive nuclei.

### Quantitative real-time PCR

Total RNA was extracted from both shPTBP1-infected groups and empty vector control-infected groups at four time points (1, 3, 7, and 14 days post-transduction) using the TRIzol RNA isolation protocol (Invitrogen, 15596026). For each reaction, 1 µg of RNA was reverse transcribed into cDNA using the Maxime RT PreMix kit (iNtron biotechnology, 25082). Subsequently, qRT–PCR was performed with Sybr Green (Thermo Fisher Scientific, 4309155) to quantify the expression of *ASCL1*,* BRN2*,* MYT1L*, and *NEUROD1*. Relative gene expression was calculated using the ΔΔCT method and normalized to *GAPDH* expression. The specific primer sequences used were: *ASCL1*: Forward 5’-CTACTCCAACGACTTGAACTCC-3’; Reverse 5’-AGTTGGTGAAGTCGAGAAGC-3’ *BRN2*: Forward 5’-GCGGATCAAACTGGGATTTAC-3’; Reverse 5’-GCACATGTTCTTGAAGCTCAG-3’ *MYT1L*: Forward 5’-CCAAACCCAAGTACCCTCAG-3’; Reverse 5’-CCAGAACCATCACAGCCAG-3’ *NEUROD1*: Forward 5’-TCCCATGTCTTCCACGTTAAG-3’; Reverse 5’-GAGAAGTTGCCATTGATGCTG-3’ *PTBP1*: Forward 5’-ATTGTCCCAGATATAGCCGTTG-3’; Reverse 5’- GCTGTCATTTCCGTTTGCTG-3’.

### Sample Preparation for multiplexed single-cell RNA sequencing

On day 14 fibroblasts transduced with shRNA targeting PTBP1 (shPTBP1) and empty vector control (shCtrl), cells were passed through a 30 μm cell strainer (Miltenyi Biotech, cat no. 130-098-458), and washed twice with cold Ca2 + and Mg2 + free 0.04% BSA/PBS (300 g, 5 min, 4℃). For multiplexing, each sample was tagged with a unique antibody-polyadenylated DNA barcode for human cells (BD Biosciences, cat no. 633781). Briefly, cells were stained with the multiplexing antibody (20 min at room temperature), and then washed 3 times with stain buffer (BD Biosciences, cat no. 554656) according to the manufacturer’s instructions. After staining, samples were gently resuspended in cold sample buffer (BD Biosciences, cat no. 633731), counted with a LUNA-FX7™ Automated Fluorescence Cell Counter (Logos biosystems), and pooled at a desired final concentration for downstream single-cell RNA sequencing.

### Single cell capture and cDNA synthesis

Following the manufacturer’s protocol (BD Biosciences), single cell capture and cDNA synthesis were performed using BD Rhapsody Express instrument. Briefly, 40,000 pooled cells from each sample were loaded in cold sample buffer onto a BD Rhapsody cartridge (cat no. 633731). After individual cell isolation, barcoded magnetic beads were added to capture and label each cell uniquely. Cell lysis was then performed, followed by retrieval of mRNA capture beads. On these beads, cDNA synthesis and subsequent Exonuclease I treatment were carried out using the BD Rhapsody cDNA kit (cat no. 633773).

### Library construction and sequencing

Single-cell RNA-seq libraries were constructed according to the mRNA Whole Transcriptome Analysis and Sample Tag Library Preparation protocol (BD Biosciences) using the BD Rhapsody WTA amplification kit (cat no. 633801). Briefly, for whole transcriptome analysis (WTA) libraries, cDNA underwent random priming and extension (RPE), followed by RPE amplification and index PCR. Sample tag libraries were prepared through nested PCR (PCR 1 & 2) and subsequent index PCR. Purified WTA and sample tag libraries were quantified by qPCR according to the qPCR Quantification Protocol Guide (KAPA) and qualified using the Agilent Technologies 4200 TapeStation. Pooled libraries were then sequenced on the HiSeq platform (Illumina), generating 150 bp paired-end reads with a targeted depth of > 20,000 reads per cell for each sample.

### Single-cell RNA-seq data analysis

Sequencing reads were processed through the BD Rhapsody WTA Analysis Pipeline in SevenBridge, generating a gene expression count matrix for downstream analysis using R v4.4.1 and Seurat v5.1.0^41^. Cells were filtered to retain those with more than 1,000 and fewer than 4,000 detected genes, and less than 20% mitochondrial gene content. After quality control, a total of 9,971 cells were retained—5,051 from the shCtrl group and 4,920 from the shPTBP1 group. Summary quality control metrics are provided in Supplementary Tables 1, and transcriptomic complexity distributions are visualized in Supplementary Fig. 1. On average, cells expressed 2,193 genes and 5,879 unique molecular identifiers (UMIs). The estimated sequencing depth per cell was approximately 61,600 reads, consistent with standard benchmarks for single-cell RNA-seq data quality. Neuronal reprogramming was independently validated in primary human fibroblast cultures derived from at least three different donors by immunofluorescence staining, and one representative sample was subsequently selected for single-cell RNA sequencing. Gene counts for cells passing quality control were normalized, log-transformed, and analyzed to identify highly variable genes. Principal component analysis (PCA) was performed on these genes to reduce dimensionality. The resulting distance matrix enabled clustering based on the Shared Nearest Neighbor (SNN) algorithm with a resolution of 0.2. Cluster visualization was performed using Uniform Manifold Approximation and Projection (UMAP)^42^. ScType function^20^ assigns cell types to clusters in single-cell RNA sequencing (scRNA-seq) data by normalizing gene expression profiles using z-scores and considering both positive and negative marker genes for various cell types. This method leverages data from the CellMarker and PanglaoDB databases to compute a cluster summary enrichment score for each cell type, derived from gene expression levels and cell type specificity scores. For this analysis, we have incorporated fibroblasts and myofibroblasts as starting material for differentiation induction into the cell type database. The cell type corresponding to the highest enrichment score is assigned to its cluster, whereas clusters with low or negative scores are classified as ‘unknown’ due to the low confidence in their annotations.

### Synaptic gene enrichment analysis

To rigorously assess synaptic gene set enrichment without circularity (“double dipping”), we applied the CountSplit method^23^. The raw UMI count matrix was split into two independent datasets (Split A and Split B) by modeling counts as a binomial process ($X_{ij} \sim \text{Binomial}(N_{ij}, 0.5)$). This ensures that the two splits are statistically independent conditional on the underlying latent gene expression. We employed a two-fold cross-validation strategy. We performed differential expression analysis (Wilcoxon rank-sum test) on the training split (e.g., Split A) to identify genes significantly upregulated in the ‘Neurons’ cluster compared to ‘Myofibroblasts’ (log2FC > 0.2, adjusted p-value < 0.05). The resulting gene list was intersected with the comprehensive SynGO ontology database^22^ to define a “Neuron-Specific Synaptic Gene Set,” thereby excluding ubiquitous structural genes shared with myofibroblasts. The gene set identified in the selection phase was used to calculate AUCell enrichment scores on the independent hold-out split (e.g., Split B). Statistical significance of the enrichment scores was evaluated solely on the validation split using pairwise Wilcoxon rank-sum tests with Bonferroni correction. This procedure was repeated by reversing the roles of Split A and Split B (Selection on B, Inference on A) to ensure robustness. All analyses were performed using Seurat (v 5.1.0) and AUCell (v 1.26.0) packages in R.

### Trajectory inference and lineage-specific analysis

For trajectory analysis, the processed Seurat object was converted into a SingleCellExperiment (SCE) object containing RNA counts, metadata and dimensionality reductions (PCA and UMAP)^43^. To simplify downstream analysis, neuronal subtypes such as ‘GABAergic neurons’ and ‘Glutamatergic neurons’ were merged under a unified ‘Neurons’ label.

Trajectory inference was performed using Slingshot with the UMAP embeddings as input. The “Fibroblasts” cluster was designated as the root population, and three terminal states - myofibroblasts, immature neurons and neurons - were assigned. This allowed the reconstruction of lineage-specific pseudotime trajectories. Cells with low quality profiles (e.g. less than 500 detected genes) were excluded before analysis.

### Trajectory-based differential expression analysis

To address the single-replicate experimental design and avoid pseudo-replication bias (“double dipping”), we implemented the Countsplit method^23^ for statistically valid trajectory-based differential expression analysis. The raw count matrix was split into two independent datasets (Split A and Split B) using binomial sampling (*p* = 0.5), such that each cell’s counts were divided into two independent partitions. We employed a bidirectional cross-validation to ensure robust gene identification. Specifically, in Path 1, trajectory inference was performed on Split A using Slingshot with UMAP embeddings, designating “Fibroblasts” as the root and identifying three terminal states (neurons, immature neurons, myofibroblasts). The pseudotime values and cell weights derived from Split A were treated as fixed covariates to fit generalized additive models (GAMs) on the independent Split B counts using tradeSeq^25^ (4 knots). Trajectory-based differential expression was assessed using the patternTest function (FDR < 0.05, Benjamini-Hochberg). In Path 2 (B→A), the procedure was reversed: trajectory inference was performed on Split B, and the resulting topology was used to test expression dynamics in Split A. The final validated gene set was defined as the intersection of significant genes identified in both cross-validation paths.

### Functional enrichment and transcription factor analysis

Validated differentially expressed genes were visualized as a heatmap and their functional relevance was explored using KEGG pathway and Gene Ontology (GO) enrichment analysis^26^. To identify transcriptional regulators governing neuronal differentiation, we screened validated genes against the DoRothEA database for high-confidence transcription factors (confidence level A-C) ^27,28^. The pseudotime expression patterns of validated transcription factors were visualized using the plotSmoothers function, highlighting lineage-specific regulatory dynamics during neuronal fate determination.

## Supplementary Information

Below is the link to the electronic supplementary material.

Supplementary Material 1

## Data Availability

The datasets generated during this study are available in the NCBI Sequence Read Archive under BioProject PRJNA1256192.

## Code availability

Analysis code is available at: https://github.com/seodojin/scRNA-seq-reprogramming-analysis.
