# Supplementary material for: Molecular characterization of directly reprogrammed neurons from human fibroblasts using single cell RNA sequencing
Source: Sci Rep. 2026 Jan 9;16:4501. doi: 10.1038/s41598-025-34688-8 (PMC12864902; doi:10.1038/s41598-025-34688-8)

**Supplementary Materials**

**Tables**

Supplementary Table 1. A summary of quality control metrics for single-cell RNA-seq analysis

Supplementary Table 2. Cell type annotation and marker gene validation

**Figures**

Supplementary Figure 1. Distribution of cell-level quality control metrics

Supplementary Figure 2. Comprehensive validation of cell type annotations through canonical markers and differential gene expression analysis.

Supplementary Figure 3. Subtype characterization of neuronal clusters
Supplementary Figure X. Validation of synaptic gene enrichment using CountSplit.

Supplementary Figure 5. NeuN immunoreactivity in differentiating SH-SY5Y cells.

**Supplementary Table 1. A summary of quality control metrics for single-cell RNA-seq analysis.**

| **Metrics** | **shCtrl** | **shPTBP1** | **Total** |
| --- | --- | --- | --- |
| Cells after QC filtering | 5051 | 4920 | 9971 |
| Mean genes per cell | 2143 | 2242 | 2193 |
| Min–Max genes per cell | 1,002–3,987 | 1,011–3,942 |  |
| Mean UMIs per cell | 5821 | 5938 | 5879 |
| Min–Max UMIs per cell | 1,567–15,772 | 1,572–15,388 |  |
| Mean % mitochondrial genes | 6.8% | 7.1% |  |
| Reads per cell (estimated) | ~61,600 | ~61,600 | 61,600 |

**Supplementary Table 2. Cell type annotation and marker gene validation.** To validate the cell type annotations, we used the *FindAllMarkers* function in Seurat (v5.1.0) to identify marker genes that are significantly upregulated in each cluster compared to all others. For each cluster, the top 10 marker genes were selected based on absolute log2 fold change and adjusted p-values. The table reports the average log2 fold change of gene expression of the target cluster compared to all other clusters (avg_log2FC), the proportion of cells expressing the gene in the target cluster and all other clusters (pct.1 and pct.2, respectively), and adjusted p-value (Benjamini–Hochberg correction, p_val_adj). Differential expression was tested using Wilcoxon rank sum test, and only positively regulated genes (only.pos = TRUE) were considered. The expression specificity of each gene was assessed by comparing its expression frequency (pct.1 vs. pct.2) and fold change values.

| Assigned_cell_type | gene | avg_log2FC | pct.1 | pct.2 | p_val_adj |
| --- | --- | --- | --- | --- | --- |
| GABAergic neurons | *CD36* | 4.539 | 0.384 | 0.026 | 2.75E-184 |
| GABAergic neurons | *GABBR2* | 4.302 | 0.296 | 0.015 | 4.48E-159 |
| GABAergic neurons | *BMP2* | 4.252 | 0.702 | 0.08 | 1.76E-267 |
| GABAergic neurons | *IL1RN* | 4.018 | 0.519 | 0.043 | 6.62E-227 |
| GABAergic neurons | *SERPINB2* | 3.974 | 0.826 | 0.186 | 4.44E-209 |
| GABAergic neurons | *WISP2* | 3.735 | 0.486 | 0.066 | 3.54E-146 |
| GABAergic neurons | *SLC16A6* | 3.618 | 0.26 | 0.025 | 3.43E-95 |
| GABAergic neurons | *FBXO32* | 3.56 | 0.528 | 0.066 | 2.05E-173 |
| GABAergic neurons | *LSAMP* | 3.367 | 0.254 | 0.028 | 6.14E-82 |
| GABAergic neurons | *SPON2* | 3.328 | 0.936 | 0.273 | 8.67E-217 |
| Glutamatergic neurons | *CYP1A1* | 7.261 | 0.289 | 0.003 | 1.03E-239 |
| Glutamatergic neurons | *HS3ST2* | 5.396 | 0.339 | 0.013 | 9.20E-221 |
| Glutamatergic neurons | *GDF15* | 5.267 | 0.691 | 0.086 | 1.43E-280 |
| Glutamatergic neurons | *GPC4* | 4.68 | 0.436 | 0.029 | 1.85E-230 |
| Glutamatergic neurons | *MAF* | 4.58 | 0.47 | 0.026 | 2.41E-268 |
| Glutamatergic neurons | *FABP3* | 4.552 | 0.366 | 0.023 | 2.03E-196 |
| Glutamatergic neurons | *MTSS1* | 4.397 | 0.475 | 0.037 | 5.27E-236 |
| Glutamatergic neurons | *HRK* | 4.189 | 0.307 | 0.024 | 1.36E-143 |
| Glutamatergic neurons | *EDIL3* | 4.155 | 0.359 | 0.022 | 1.19E-191 |
| Glutamatergic neurons | *IL33* | 4.129 | 0.439 | 0.041 | 4.30E-193 |
| Myofibroblasts | *ANGPT1* | 1.661 | 0.508 | 0.247 | 1.24E-87 |
| Myofibroblasts | *OXTR* | 1.553 | 0.325 | 0.179 | 4.20E-32 |
| Myofibroblasts | *TNFRSF11B* | 1.441 | 0.604 | 0.377 | 2.66E-69 |
| Myofibroblasts | *TENM2* | 1.414 | 0.329 | 0.16 | 9.42E-42 |
| Myofibroblasts | *COL8A1* | 1.298 | 0.659 | 0.5 | 2.35E-54 |
| Myofibroblasts | *GREM1* | 1.266 | 0.994 | 0.945 | 9.44E-268 |
| Myofibroblasts | *PSG5* | 1.246 | 0.348 | 0.2 | 7.25E-29 |
| Myofibroblasts | *CXCL6* | 1.211 | 0.521 | 0.353 | 1.62E-35 |
| Myofibroblasts | *F3* | 1.179 | 0.617 | 0.392 | 1.80E-61 |
| Myofibroblasts | *WNT5A* | 1.164 | 0.515 | 0.326 | 3.75E-47 |
| Immature neurons | *CDCA8* | 4.312 | 0.377 | 0.027 | 6.47E-208 |
| Immature neurons | *UBE2C* | 4.142 | 0.482 | 0.052 | 1.42E-258 |
| Immature neurons | *NCAPH* | 4.073 | 0.267 | 0.019 | 3.02E-137 |
| Immature neurons | *CKAP2L* | 4.063 | 0.407 | 0.033 | 7.56E-222 |
| Immature neurons | *NUF2* | 4 | 0.268 | 0.021 | 1.80E-134 |
| Immature neurons | *KIF2C* | 3.909 | 0.379 | 0.034 | 3.90E-198 |
| Immature neurons | *KIF11* | 3.902 | 0.281 | 0.021 | 3.25E-143 |
| Immature neurons | *KIFC1* | 3.897 | 0.424 | 0.037 | 1.49E-227 |
| Immature neurons | *PLK1* | 3.896 | 0.45 | 0.05 | 1.52E-234 |
| Immature neurons | *HJURP* | 3.871 | 0.364 | 0.03 | 2.88E-190 |
| Fibroblasts | *PRG4* | 5.678 | 0.285 | 0.013 | 1.59E-148 |
| Fibroblasts | *SFRP4* | 5.415 | 0.259 | 0.012 | 1.79E-130 |
| Fibroblasts | *CYTL1* | 5.234 | 0.344 | 0.014 | 2.89E-187 |
| Fibroblasts | *EFEMP1* | 5.102 | 0.811 | 0.069 | 0.00E+00 |
| Fibroblasts | *LIF* | 4.591 | 0.259 | 0.014 | 1.28E-120 |
| Fibroblasts | *NDUFA4L2* | 4.549 | 0.519 | 0.074 | 4.65E-132 |
| Fibroblasts | *PTX3* | 4.287 | 0.741 | 0.21 | 7.84E-117 |
| Fibroblasts | *PTGIS* | 4.046 | 0.696 | 0.083 | 2.35E-217 |
| Fibroblasts | *PTHLH* | 4.017 | 0.363 | 0.044 | 1.52E-96 |
| Fibroblasts | *PENK* | 3.991 | 0.404 | 0.043 | 2.07E-122 |

**Supplementary Figure 1. Distribution of cell-level quality control metrics.** Violin plots illustrate the distribution of the number of detected genes per cell (nFeature_RNA), total UMI counts per cell (nCount_RNA), and the percentage of mitochondrial gene expression (percent.mt) in the shCtrl and shPTBP1 groups. These values were used to apply filtering thresholds (>1,000 and <4,000 genes per cell and <20% mitochondrial content), ensuring high-quality data for downstream analysis.


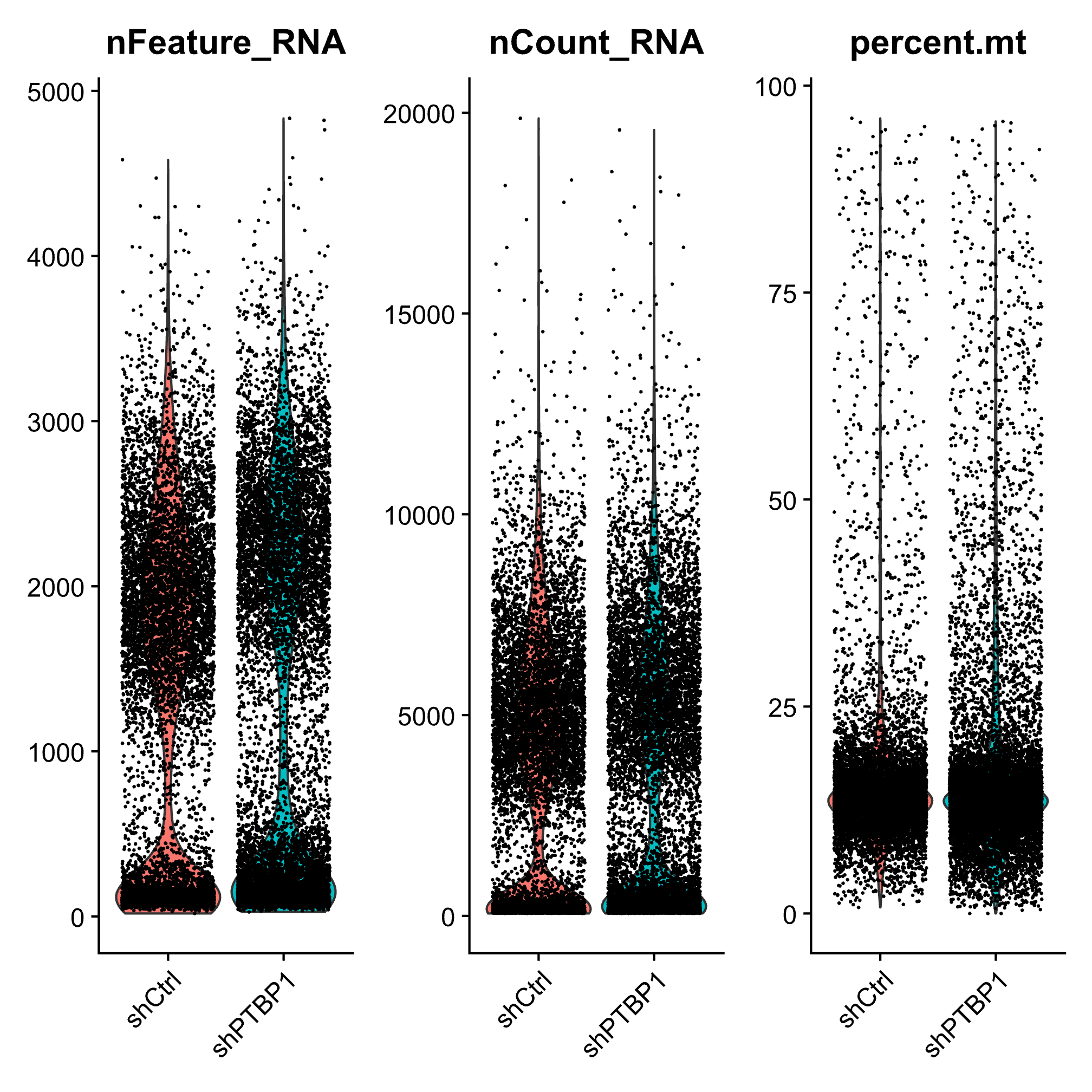


**Supplementary Figure 2. Comprehensive validation of cell type annotations through canonical markers and differential gene expression analysis.** (a) Violin plots showing expression of canonical marker genes across cell types. *TUBA1C, ACTA2*, and *COL1A1* is a marker of immature neurons, myofibroblasts and fibroblasts, respectively; *STMN2* serves as a pan-neuronal maturation marker expressed in both glutamatergic and GABAergic neurons; *GRIN2A* and *GABBR2* distinguish glutamatergic and GABAergic neuronal subtypes, respectively. All markers show statistically significant differential expression (Kruskal-Wallis test, *p* < 0.001), validating our cell type annotations. (b) Heatmap of the top five up-regulated genes per cluster in the combined dataset (Fibroblasts, GABAergic neurons, Glutamatergic neurons, Immature neurons, Myofibroblasts). Differential genes were identified using Seurat v5.1.0 *FindAllMarkers* (Wilcoxon rank-sum; only.pos=TRUE; min.pct=0.25; logfc.threshold=0.25) and retained at BH-adjusted p<0.05. Values show row-wise scaled (z-score) expression from log-normalized data; columns are cells grouped by cluster (down-sampled to ≤300 cells/cluster for visualization).

(a)


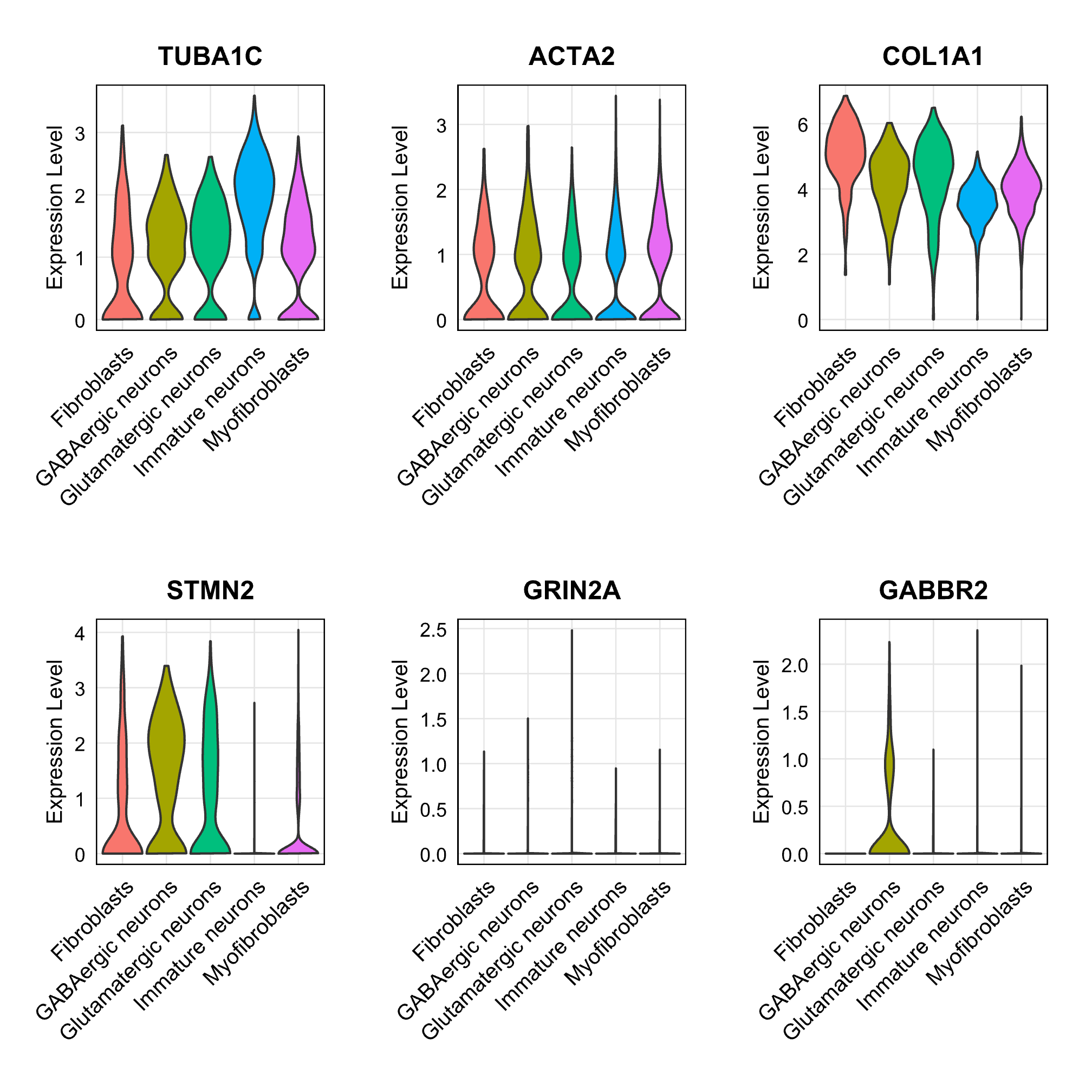


(b)


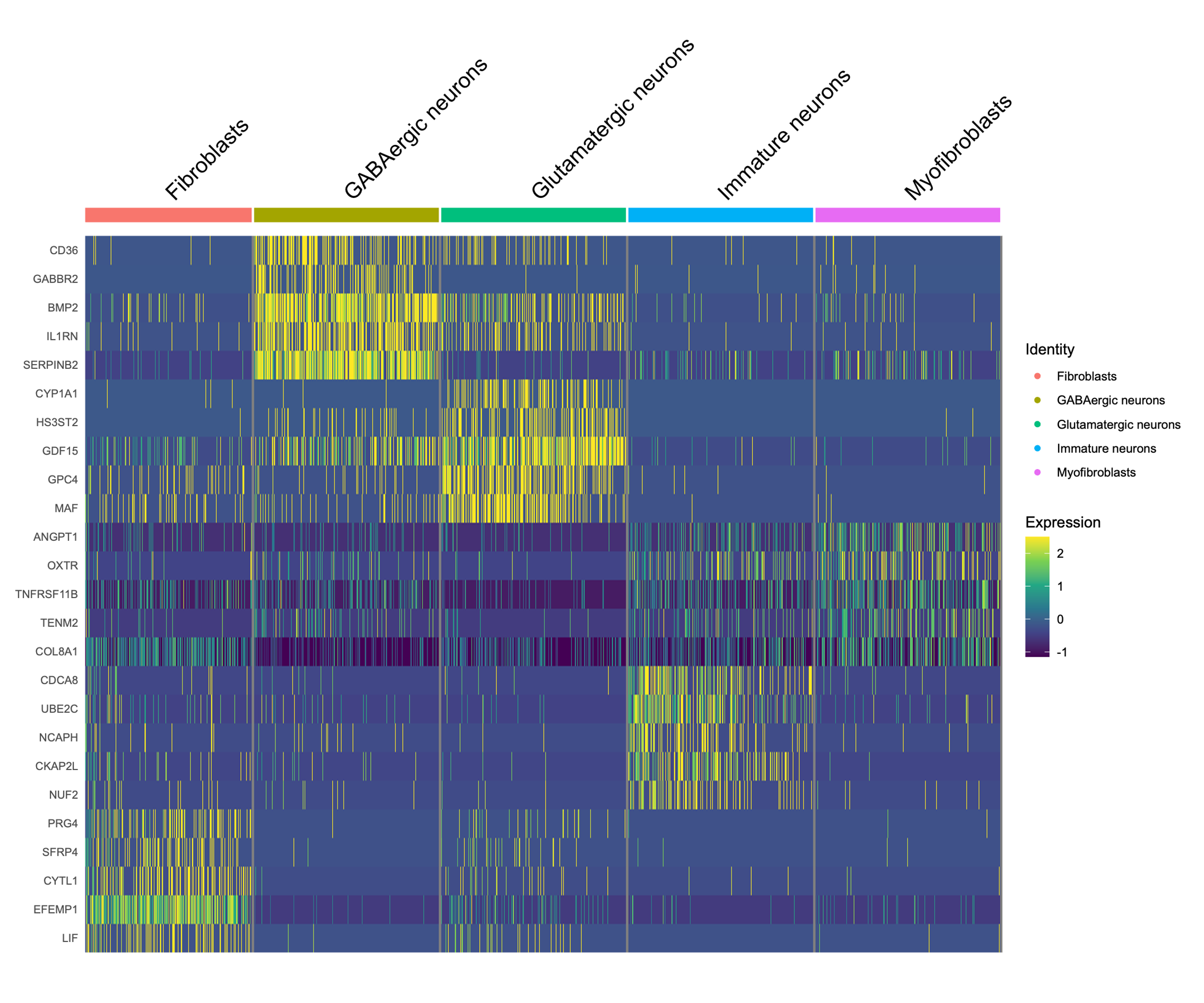


**Supplementary Figure 3. Subtype characterization of neuronal clusters.** (a) UMAP feature plots showing the expression of canonical neuronal subtype markers. *GRIN2A* (glutamatergic) and *GABBR2* (GABAergic) are expressed in distinct neuronal subclusters (red circles). In contrast, *ACHE* (cholinergic) and *TH* (dopaminergic) show minimal or absent expression. (b) Immunofluorescence staining for subtype-specific neuronal markers. Cells were stained with antibodies against subtype-specific neuronal markers: **vGlut1** (glutamatergic), **GABA-T** (GABAergic), **ChAT** (cholinergic), and **TH** (dopaminergic), along with **MAP2** (neuronal marker, green) and **DAPI** (nuclei, blue). **vGlut1- and GABA-T-positive cells (red arrows)** confirm the presence of **glutamatergic** and **GABAergic** neurons, respectively. **ChAT** and **TH** signals were not detected, indicating the absence of detectable cholinergic and dopaminergic differentiation under these conditions. Scale bar = 50 μm.


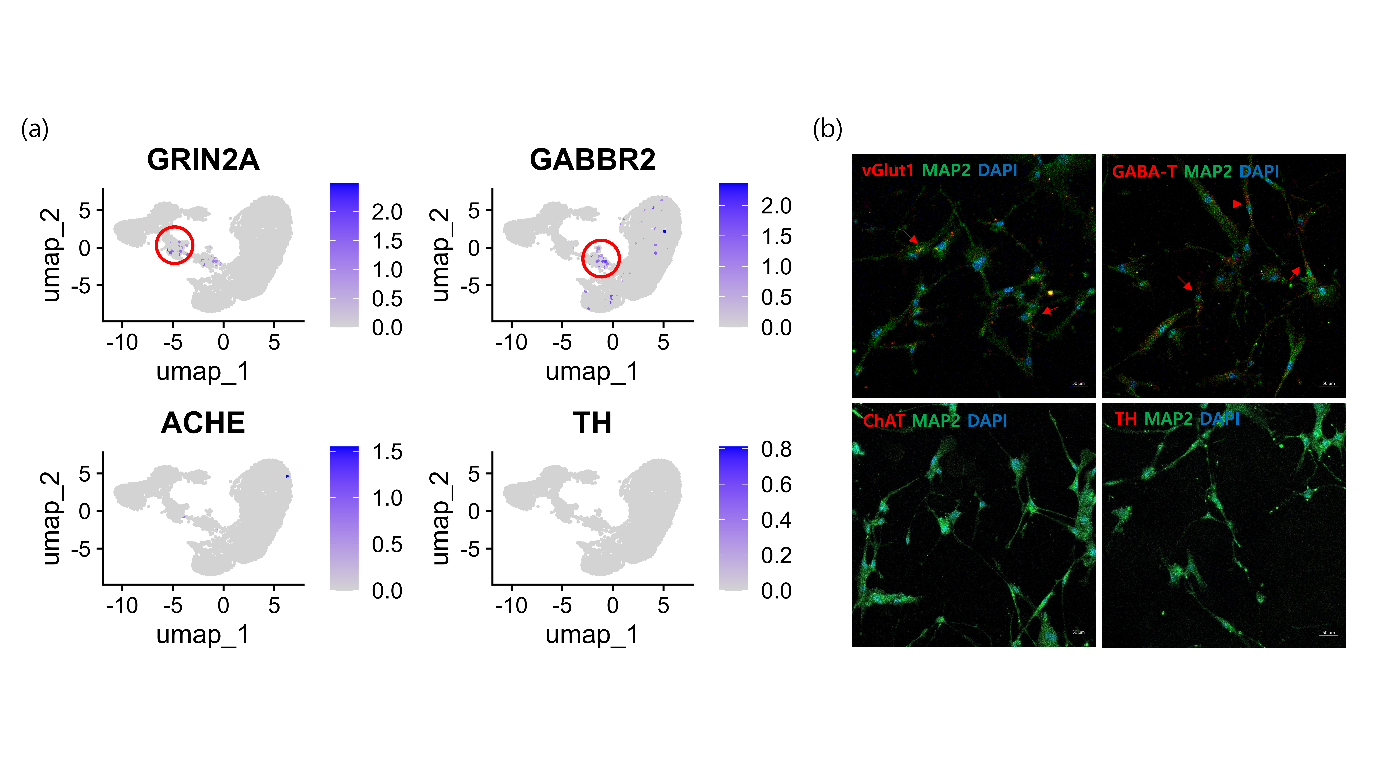


**Supplementary Figure X. Validation of synaptic gene enrichment using CountSplit.** (a) Violin plots showing AUCell enrichment scores for neuron-specific SynGO genes. To avoid selection bias, genes were selected based on differential expression in Split A (Neurons vs. Myofibroblasts, FDR < 0.05), while scores were calculated and tested on the independent Split B. (b) Cross-validation results showing the reverse analysis (Selection in Split B, Scoring in Split A). Note that in both independent validations, mature neurons show significant enrichment of synaptic programs compared to myofibroblasts (** p < 0.001, Wilcoxon rank-sum test), confirming the specificity of the synaptic signature.


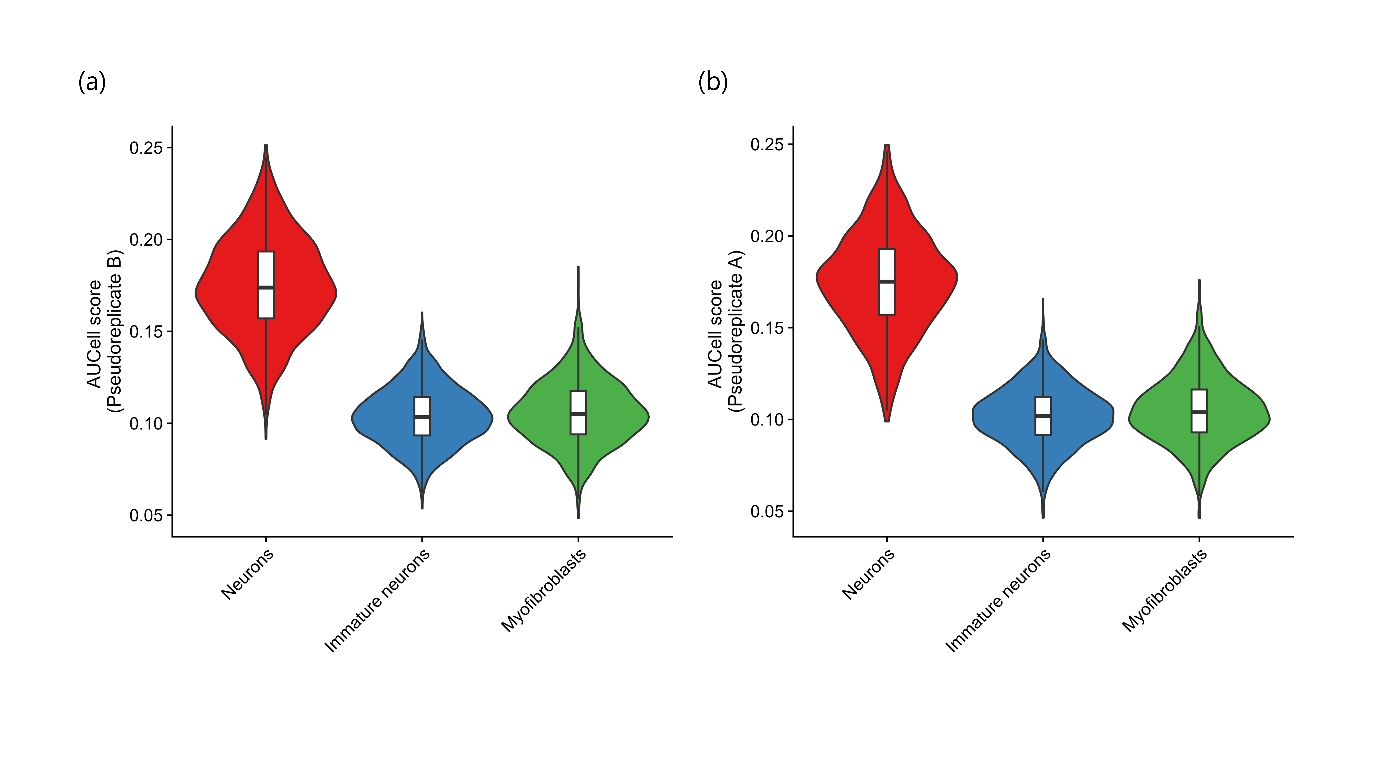


**Supplementary Figure 5. NeuN immunoreactivity in differentiating SH-SY5Y cells.** Representative images showing widespread both nuclear and cytoplasmic NeuN expression (green), a characteristic feature in developmental contexts (Lundgaard et al., 2015). Anti-NeuN antibody, Sigma-Aldrich MAB377 (clone A60). Nuclei: DAPI (blue). Scale bar, 50 µm.


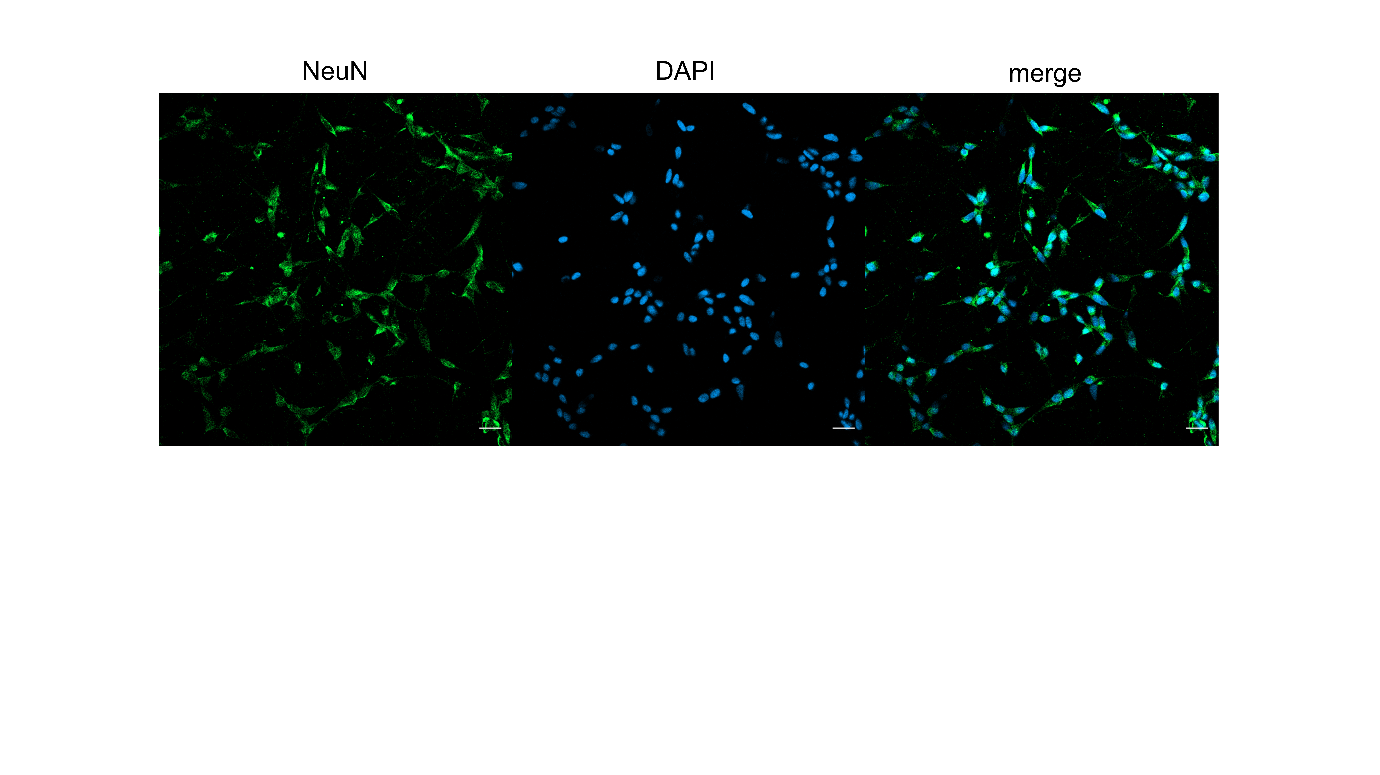

Supplement: Supplementary file 1 — Supplementary Material 1 [file 41598_2025_34688_MOESM1_ESM.docx]
